# Factors associated with afebrile presentation and delayed defervescence of bacterial meningitis in children under 3 years of age: a multi-centre retrospective analysis

**DOI:** 10.1186/s12887-023-04179-8

**Published:** 2023-09-18

**Authors:** Lin He, Haijing Li, Zhigang Zhang, Hejia Ge, Hongwei Wang, Mengquan Zhu, Zhiwei Xu, Jiening Zhang, Sheng Fang, Chuanze Hu, Lijun Qian, Huifang Xu, Yinna Yao, Shengfu Yuan, Jiajun Zhu, Chaosheng Lu, Jishan Zheng, Junsheng Li, Qi Jiang, Huiqing Xu, Lihua Chen, Shiqiang Shang, Yinghu Chen

**Affiliations:** 1 Department of Infectious Diseases, The Children’s Hospital, Zhejiang University School of Medicine, National Clinical Research Center For Child Health, Hangzhou, China; 2 Zhejiang University School of Medicine, Hangzhou, China; 3 Department of Pediatric, Shaoxing Maternity and Child Health Care Hospital, Shaoxing, China; 4 Department of Pediatric, Taizhou Central Hospital (Taizhou University Hospital), Taizhou, China; 5 Department of Pediatric, The Second Affiliated Hospital of Jiaxing University, Jiaxing, China; 6 Department of Pediatric, Shaoxing Keqiao Women and Children’s Hospital, Shaoxing, China; 7 Department of Pediatric, The Second Affiliated Hospital and Yuying Children’s Hospital of Wenzhou Medical University, Wenzhou, China; 8 Department of Pediatric, Jiaxing Maternity and Child Health Care Hospital, Jiaxing, China; 9 Department of Pediatric, Taizhou Hospital of Zhejiang Province Affiliated to Wenzhou Medical University, Taizhou, China; 10 Department of Pediatric, Jinhua Maternal and Child Health Care Hospital, Jinhua, China; 11 Department of Pediatric, Lishui Central Hospital and Fifth Affiliated Hospital of Wenzhou Medical College, Lishui, China; 12 Department of Pediatric, Lanxi People’s Hospital, Lanxi, China; 13 Department of Pediatric, Zhuji People’s Hospital of Zhejiang Province, Zhuji, China; 14 Department of Pediatric, Yuyao People’s Hospital, Yuyao, China; 15 Division of Neonatology, Women’s Hospital, Zhejiang University School of Medicine, Hangzhou, China; 16 Department of Pediatric, The First Affiliated Hospital of Wenzhou Medical University, Wenzhou, China; 17 Department of Pediatric, Ningbo Women and Children Hospital, Ningbo, China; 18 Department of Pediatric, Lishui Maternity and Child Health Care Hospital, Lishui, China; 19 Department of Pediatric, Huzhou Maternity and Child Health Care Hospital, Huzhou, China; 20 Division of Neonatology, The Children’s Hospital, Zhejiang University School of Medicine, National Clinical Research Center For Child Health, Hangzhou, China; 21 Department of Clinical Laboratory, The Children’s Hospital, Zhejiang University School of Medicine, National Clinical Research Center For Child Health, Hangzhou, China

**Keywords:** Fever, Defervescence time, Bacterial meningitis, Children

## Abstract

**Background:**

This multi-center study aimed to identify factors affecting fever and delayed defervescence in bacterial meningitis (BM) patients under 3 years of age because of the variability of fever in this patient population.

**Methods:**

Only BM patients under 3 years treated at 49 centers in China from November 2018 to end-April 2021 were included in the study. Univariate and multivariate logistic regression analyses were performed to determine factors associated with afebrile presentation and fever of delayed defervescence.

**Results:**

A total of 863 BM patients under 3 years were included in the study. Coagulase negative staphylococcus was associated with afebrile presentation (OR = 1.176), while septicaemia and ear-nose-throat infections were associated with fever (*P* < 0.05). The patients with fever were assigned into early and delayed defervescence groups based on defervescence time (less than and more than or equal to one week). Furthermore, Streptococcus agalactiae meningitis (OR = 1.124), concomitant gastrointestinal infection (OR = 1.276), encephalomalacia (or = 1.339), and subdural effusion (OR = 1.454) were independently associated with delayed defervescence (all *P* < 0.05).

**Conclusions:**

The findings can aid in the efficient utilization of fever in auxiliary diagnosis and evaluating the condition of the disease.

**Supplementary Information:**

The online version contains supplementary material available at 10.1186/s12887-023-04179-8.

## Background

Bacterial meningitis (BM) is one of the common life-threatening diseases among pediatric patients, often leading to serious sequelae [1]. Despite that increasing vaccination rates have reduced the incidence of BM, the burden of BM in younger children with BM remains substantial, especially in developing countries such as China. The burden of BM in younger children in China is about 6.95–22.3 cases per 100,000 children < 5 years old [2]. Timely diagnosis and reasonable adjustment of therapy according to condition are crucial for the prognosis of BM [3]. Besides, common clinical manifestations of BM are crucial in diagnosing and judging the severity of the condition since it is achieved using fever in the clinical course of childhood pneumonia [4]. However, the presence or absence and presentation of clinical symptoms, such as fever and nuchal rigidity, are highly variable among younger children with BM, thus limiting BM diagnosis or condition judgment [5, 6].

Fever is one of the major clinical manifestations of childhood BM, and has been incorporated into multiple guidelines for BM clinical practice as an auxiliary diagnostic basis [5, 7]. Nonetheless, fever greatly varies among individuals with BM, especially in young children under 3 years of age, thus delaying the treatment process [3, 8]. Previous studies showed that BM patients under 3 years often have temperature instability, manifested as fever, normothermia, or hypothermia, possibly due to immaturity of cellular and humoral immunity [9–11]. Some studies have investigated factors that independently influence fever in pediatric BM patients. Also, many studies have judged BM condition in an older pediatric age group using defervescence time as an important reference [12]. Delayed defervescence is associated with subdural effusion, one of the serious complications of BM [13]. Therefore, other factors affecting defervescence time should be explored to enhance the clinical utility of the defervescence time in prognosticating BM outcomes.

Although most studies have only stated that fever is variable in younger children with BM, the detailed causes and impact factors are unknown, thus limiting the rational use of fever in diagnosing BM in younger children or judging the patient’s condition. This multi-centre study aimed to identify factors that are independently associated with fever and defervescence time in BM patients under 3 years of age.

## Methods

### Patients

Only pediatric BM inpatients under 3 years of age in 49 tertiary hospitals, including specialist pediatric and general hospitals in Zhejiang province, China (from November 2018 to May 2021) were included in this multi-center retrospective study. The 49 tertiary hospitals serve more than 8.6 million children in the region. Data collection, management, and analysis were performed by doctors from the departments of infectious diseases at Children’s Hospital of Zhejiang University, National Clinical Research Center for Child Health (China) from May 2021 to March 2022. This study was approved by the Ethics Committee of the Children’s Hospital, Zhejiang University School of Medicine, National Clinical Research Center for Child Health (reference number 2019-IRB-094). The informed consent requirement was waived since this is a retrospective study, where the patient data are anonymous.

### Inclusion and exclusion criteria

Inclusion criteria were: BM patients aged less than 3 years and BM patients mainly diagnosed based on clinical symptoms, examination of cerebrospinal fluid (CSF), and clinical judgement. Furthermore inclusion criteria were: Neonatal patients with sepsis, irritability, feeding difficulty, respiratory failure, mottled skin, poor muscle tone, seizures, or fever were classified as suspected neonatal BM cases. Children older than one month of age with impaired mental status, fever, meningeal irritation, or headache were classified as suspected non-neonatal BM cases. BM in neonatal patients was diagnosed based on a positive CSF culture or negative CSF cultures and CSF white cell count > 21 cells/mm3 [14, 15]. BM diagnosis in non-neonatal patients was based on a positive CSF cultures or at least one of the following in CSF white cell count > 2000 cells/uL, CSF polymorphonuclear cells > 1180/uL, CSF protein > 2.2 g/L, CSF glucose < 1.9 mmol/L, and CSF/serum glucose ratio > 0.23 [16]. Exclusion criteria: Patients diagnosed with viral meningitis, tuberculous or fungal meningitis, and patients with missing clinical data.

### Groupings and definitions

The BM patients were divided into a non-fever group and a fever group based on whether fever occurred during the course of the disease or not. The BM patients with fever were further subgrouped into an early defervescence group (defervescence time < 1 week) and a delayed defervescence group (defervescence time ≥ 1 week) based on defervescence time.

The treatment regimens of each patient were compared with the European Society for Clinical Microbiology and Infectious Diseases (ESCMID) guideline (an authoritative guideline for BM). Variables regarding patient treatment regimens were subdivided into whether the antibiotics met the guideline, whether the antibiotic course was adequate, and whether the adjuvant steroid therapy met the guideline based on guideline recommendations [5]. Notably, judgement of adequacy of antibiotic regimen was made in the context of compliance of the treatment regimen with the guidelines.

### Data collection

The clinical data, such as demographic data, complications with other foci of infection, underlying diseases, cerebral complications (identified by cranial ultrasound, computed tomography, or magnetic resonance imaging), bacterial culture results, antibiotics therapy, and adjuvant steroid regimen data, of all patients were independently collected and carefully reviewed by two researchers. The above information was selected on the basis of potential relevance as judged by previous studies and clinical practice. Any discrepancies were resolved by discussion and consulting a third researcher.

Notably, some children had coagulase-negative staphylococcal (CoNS) infections. CoNS are usually found in superficial areas such as the skin, and such isolates may represent contaminating organisms and thus patients with meningitis (CoNS is the potential pathogen) were strictly assessed as follows: Patients with positive bacterial cultures for CoNS were included if the bacteria were cultured in both blood and cerebrospinal fluid and the drug sensitivity test was consistent, or if the cerebrospinal fluid was retested for the same bacteria and the drug sensitivity was consistent, and if the clinical features and the rest of the tests met the criteria for bacterial meningitis. However, these patients were excluded if CoNS were only culture-positive in the cerebrospinal fluid and the bacterium was not cultured in the blood, while the child's clinical features and other laboratory indicators did not meet the criteria for bacterial meningitis. In addition, this group of patients was excluded if the clinical features met the clinical diagnostic criteria for chemoencephalitis and a single specimen in the cerebrospinal fluid or blood was cultured for CoNS, but the culture was not retested for the same pathogen or if the retest was for the same pathogen but the drug sensitivity was inconsistent. Nine such children were excluded during the study period, and the specific information is shown in the Supplementary file.

### Statistical analysis

Statistical analysis was performed using Statistical Package for Social Science (SPSS) version 22.0 for Windows (IBM, USA). Quantitative variables were expressed as mean and 95% confidence interval (95%CI), while categorical variables were expressed as counts and percentages. The categorical variables in univariate analysis were assessed using Pearson's Chi-square test. The odds ratios (OR) and 95% CI were also calculated. Quantitative variables were analyzed using two-tailed t-tests or Wilcoxon rank-sum tests where appropriate. Multivariate logistic regression analysis was used to identify independent influencing factors between different groups. *P* < 0.05 was considered a significant difference.

## Results

### Patient inclusion and characteristics

**Fig. 1:**
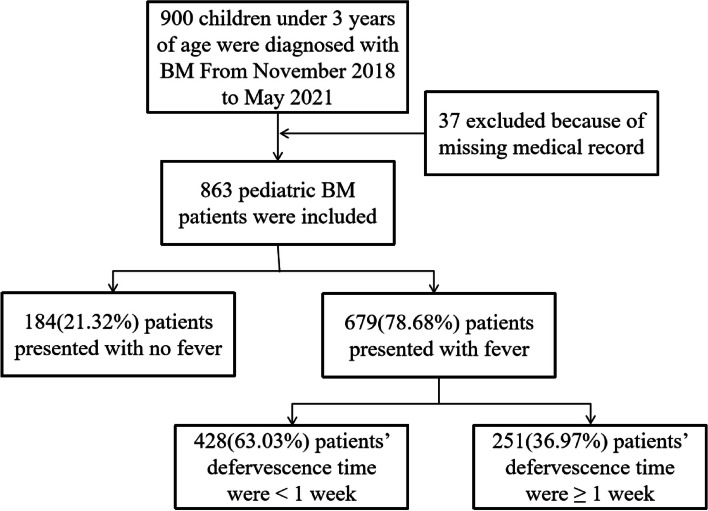
Flowchart of pediatric BM patients selection, grouping and subgrouping

Only 863 (558 (64.7%) males and 305 (35.3%) females) of 900 pediatric BM patients under 3 years diagnosed at the 49 tertiary hospitals were included in this study. The 37 excluded patients did not have complete medical records. A flowchart of patient selection and grouping is presented in Fig. 1. The average age of the enrolled patient was 2.90 months (95% CI, 2.61,3.20 months), ranging from newborn to 3 years old patients (less than 36 months). A total of 197 patients (197/863,22.8%) had underlying diseases, of which 125 cases (125/197,63.5%) had congenital heart diseases, 16 cases (16/197,8.1%) had anaemia, 13 cases (13/197,6.6%) had chronic pulmonary diseases, and 100 cases (100/197,50.8%) had other underlying conditions. Furthermore, 747 patients (747/863,86.6%) had other foci of infection, including 644 cases (644/747,86.2%) of septicemia, 168 cases (168/747,22.5%) of respiratory infection, 66 cases (66/747,8.8%) of gastrointestinal infection, 51 cases (51/747,6.8%) of ear-nose-throat (ENT) infection, 50 cases (50/747,6.7%) of urinary infection, 6 cases (6/747,0.8%) of skin infection, and 15 cases (15/747,2.0%) of infection at other sites.

All pediatric BM patients underwent lumbar puncture for CSF culture, and blood culture results of 678 patients (78.6%) were obtained. A total of 226 cases (226/86326.2%) had positive bacterial cultures from CSF or blood, including *Escherichia coli* (68/226,30.1%), *Streptococcus agalactiae* (64/226,28.3%), coagulase-negative *Staphylococci* (42/226,18.6%), *Staphylococcus aureus* (10/226,4.4%), *Streptococcus pneumoniae* (6/226,2.7%), other gram-positive bacteria (16 cases,1.9%) and other gram-negative bacteria (25/226, 11.1%). Thirty-one cases (31/226,13.7%) were infected with two or more bacterial species. Imaging results and cranial ultrasound (109/863,12.6%) showed that 275 cases (275/863,31.9%) had cerebral complications, of which 181 cases (181/863,21.0%) had cerebrovascular complications, such as hemorrhage or infarction, 67 cases (67/181,37.0%) had subdural effusion, 71 cases (71/181,39.2%) had hydrocephalus, 43 cases (43/181,23.8%) had encephalomalacia, 23 cases (23/181,12.7%) had cerebral edema, 12 cases (12/181,6.6%) had ependymitis, 4 cases (4/181,2.2%) had cerebral abscess, and 18 cases (18/181,9.9%) had other cerebral complications. The median time to start antibiotic therapy was 1.0 (0.5,1.5) day after admission. Notably, the regimens used to treat the infection in 526 (61.0%) cases complied with ESCMID guidance, and were adequate in 415 (48.1%) cases. A total of 83 cases (9.6%) were treated with steroids during the treatment, but steroid therapy of only 44 (5.1%) cases ha complied with ESCMID guidelines.

### Factors associated with BM in the absence of fever

A total of 184 (21.3%) BM patients did not have a fever. Univariate analysis showed that the absence of fever was associated with neonatal age group, underlying co-morbidities, cerebrovascular complications, infection with coagulase-negative Staphylococci or other gram-positive bacteria, and isolation of more than one bacterial species on CSF culture (all *P* < 0.05; Table 1). The significant variables in univariate analysis were selected for multivariate analysis, and results showed that isolation of coagulase-negative Staphylococci (OR 1.176; 95% CI, 1.046–1.322) was independently associated with a lack of fever. Septicaemia and ENT infection were independently associated with fever (Table 2).

**Table 1:** Comparison of demographic, etiological, clinical and treatment data between non-fever group and fever groups

	Non-fever group (*N* = 184)	Fever group (*N* = 679)	*X*^2^/Z	*P*	OR (95%CI)
Male (N, %)	112(60.87%)	446(65.68%)	1.469	0.226	0.813(0.581,1.137)
Complicated with other foci of infection					
**Septicaemia (N, %)**	**118(64.13%)**	**526(77.47%)**	**13.597**	** < 0.001**^**a**^	**0.520(0.366,0.739)**
Respiratory infection (N, %)	41(22.28%)	127(18.70%)	1.183	0.277	1.246(0.838,1.854)
**ENT infection (N, %)**	**2(1.09%)**	**49(7.22%)**	**9.782**	**0.002**^**a**^	**0.141(0.034,0.587)**
Urinary infection (N, %)	11(5.98%)	39(5.74%)	0.015	0.904	1.043(0.523,2.080)
Gastrointestinal infection (N, %)	9(4.89%)	57(8.39%)	2.516	0.113	0.561(0.272,1.156)
Skin infection (N, %)	0(0)	6(0.88%)	1.637	0.201	-
Other sites of infection (N, %)	3(1.63%)	12(1.77%)	< 0.001	1.000	0.921(0.257,3.299)
Underlying diseases					
Chronic nervous system diseases (N, %)	0(0)	4(0.59%)	0.186	0.666	-
**Chronic pulmonary diseases (N, %)**	**6(3.26%)**	**7(1.03%)**	**4.852**	**0.028**^**a**^	**3.236(1.074,9.749)**
Congenital heart diseases (N, %)	26(14.13%)	99(14.58%)	0.024	0.878	0.964(0.605,1.537)
Brain malformations (N, %)	1(0.54%)	0(0)	0.491	0.484	-
Immunodeficiency disease (N, %)	5(2.72%)	7(1.03%)	1.899	0.168	2.682(0.841,8.549)
Malnourished (N, %)	2(1.09%)	2(0.29%)	0.627	0.428	3.720(0.520,26.588)
Abnormal renal function (N, %)	2(1.09%)	9(1.33%)	< 0.001	1.000	0.818(0.175,3.819)
Anaemia (N, %)	5(2.72%)	11(1.62%)	0.450	0.502	1.696(0.582,4.945)
**Other underlying diseases (N, %)**	**22(11.96%)**	**46(6.77%)**	**5.355**	**0.021**^**a**^	**1.869(1.093,3.196)**
Cerebral complications					
Hydrocephalus (N, %)	16(8.70%)	55(8.10%)	0.068	0.794	1.081(0.604,1.934)
Ependymitis (N, %)	3(1.63%)	9(1.33%)	< 0.001	1.000	1.234(0.331,4.605)
**Cerebrovascular complications (N, %)**	**50(27.17%)**	**131(19.29%)**	**5.425**	**0.020**^**a**^	**1.561(1.071,2.275)**
Cerebral abscess (N, %)	0(0)	4(0.59%)	1.089	0.297	-
Encephalomalacia (N, %)	12(6.52%)	31(4.57%)	1.170	0.279	1.458(0.734,2.900)
Subdural effusion (N, %)	12(6.52%)	55(8.10%)	0.504	0.478	0.792(0.415,1.512)
Cerebral edema (N, %)	4(2.17%)	19(2.80%)	0.043	0.835	0.772(0.259,2.298)
Other cerebral complications (N, %)	5(2.72%)	13(1.91%)	0.148	0.700	1.431(0.504,4.067)
Bacterial cultures					
Escherichia coli (N, %)	12(6.52%)	56(8.25%)	0.594	0.441	0.776(0.407,1.481)
Streptococcus agalactiae (N, %)	10(5.43%)	54(7.95%)	1.337	0.248	0.665(0.332,1.333)
**Coagulase-negative Staphylococci (N, %)**	**17(9.24%)**	**25(3.68%)**	**9.657**	**0.002**^**a**^	**2.663(1.405,5.046)**
Streptococcus pneumoniae (N, %)	1(0.54%)	5(0.74%)	< 0.001	1.000	0.737(0.086,6.344)
Staphylococcus aureus (N, %)	1(0.54%)	9(1.33%)	0.241	0.624	0.407(0.051,3.232)
**Other Gram-positive bacteria (N, %)**	**9(4.89%)**	**7(1.03%)**	**11.856**	**0.001**^**a**^	**4.937(1.813,13.443)**
Other Gram-negative bacteria (N, %)	6(3.26%)	19(2.80%)	0.110	0.740	1.171(0.461,2.976)
**Bacterial species ≥ 2 (N, %)**	**14(7.61%)**	**17(2.50%)**	**10.894**	**0.001**^**a**^	**3.207(1.550,6.636)**
Time to start antibiotic therapy (day)	2.4(1.3,3.5)	0.7(0.5,0.8)	-1.187	0.235	-
Antibiotic regimens complied with guideline (N, %)	105(57.06%)	421(62.00%)	1.483	0.223	0.815(0.585,1.134)
Adequate antibiotic treatment course (N, %)	81(64.80%)	334(71.98%)	2.441	0.118	0.717(0.471,1.090)
**Adjuvant steroid regimens complied with guideline** (N, %)	**3(1.63%)**	**41(6.04%)**	**5.813**	**0.016**^**a**^	**0.258(0.079,0.843)**

*BM* bacterial meningitis; OR (95%CI): odds ratio and 95% confidence interval; ^**a**^* P* < 0.05

**Table 2:** Factors independently associate with an afebrile presentation of BM in children under 3 years

	B	S.E	Wald	*P*	Exp (B)	95%Exp (B)	
						Lower limit	Upper limit
**Septicaemia**	**-0.095**	**0.024**	**16.429**	** < 0.001**^**a**^	**0.909**	**0.868**	**0.952**
**ENT infection**	**-0.413**	**0.148**	**7.828**	**0.005**^**a**^	**0.661**	**0.495**	**0.884**
Chronic pulmonary diseases	0.103	0.086	1.458	0.227	1.109	0.938	1.311
Other underlying diseases	0.065	0.037	3.124	0.077	1.067	0.993	1.146
Cerebrovascular complications	0.046	0.035	1.757	0.185	1.047	0.978	1.120
**Coagulase-negative Staphylococci**	**0.162**	**0.060**	**7.347**	**0.007**^**a**^	**1.176**	**1.046**	**1.322**
Other Gram-positive bacteria	0.133	0.079	2.788	0.095	1.142	0.977	1.334
Bacterial species ≥ 2	0.080	0.061	1.755	0.185	1.084	0.962	1.220
Adjuvant steroid regimens complied with guideline	-0.150	0.077	3.771	0.052	0.861	0.740	1.001

*BM* bacterial meningitis; ^**a**^*P* < 0.05

### Factors associated with defervescence time

A total of 251 (37.0%) of 679 patients with fever experienced delayed defervescence during management. Univariate analysis showed gastrointestinal infection, chronic pulmonary disease, congenital heart disease, cerebrovascular complications, intracranial complications (hydrocephalus, ependymitis, encephalomalacia, or subdural effusion), *Streptococcus agalactiae* and antibiotic regimens that complied with guidelines were significantly associated with delayed defervescence (Table 3). Furthermore, univariate analysis showed that infant age category and shorter time to initiation of antibiotic therapy were associated with defervescence time more than or equal to one week (*P* < 0.05). Also, the significant variables in univariate analysis were selected for multivariate analysis, and results showed that gastrointestinal infection, encephalomalacia, cerebrovascular complications, subdural effusion, and *Streptococcus agalactiae* were independently associated with delayed defervescence (more than or equal to one week) (Table 4).

**Table 3:** Comparison of demographic, etiological, clinical and treatment data between delayed and early defervescence group

	Delayed defervescence group (*N* = 251)	Early defervescence group (*N* = 428)	*X*^2^/Z	*P*	OR (95%CI)
Male (N, %)	167 (66.53%)	279 (65.19%)	0.127	0.721	1.062(0.764,1.475)
Complicated with other foci of infection					
Septicaemia (N, %)	201(80.08%)	325(75.93%)	1.557	0.212	1.274(0.871,1.865)
Respiratory infection (N, %)	51(20.32%)	76(17.76%)	0.683	0.409	1.181(0.796,1.753)
ENT infection (N, %)	15(5.98%)	34(7.94%)	0.915	0.339	0.737(0.393,1.381)
Urinary infection (N, %)	13(5.18%)	26(6.07%)	0.234	0.628	0.845(0.426,1.675)
**Gastrointestinal infection (N, %)**	**30(11.95%)**	**27(6.31%)**	**6.553**	**0.010**^**a**^	**2.016(1.169,3.478)**
Skin infection (N, %)	1(0.40%)	5(1.17%)	0.372	0.542	0.338(0.039,2.913)
Other sites of infection (N, %)	7(2.79%)	5(1.17%)	1.551	0.213	2.427(0.762,7.730)
Underlying diseases					
Chronic nervous system diseases (N, %)	1(0.40%)	3(0.70%)	< 0.001	1.000	0.567(0.059,5.477)
**Chronic pulmonary diseases (N, %)**	**6(2.39%)**	**1(0.23%)**	**5.254**	**0.022**^**a**^	**10.457(1.252,87.366)**
**Congenital heart diseases** (N, %)	**51(20.32%)**	**48(11.21%)**	**10.529**	**0.001**^**a**^	**2.019(1.314,3.103)**
Brain malformations (N, %)	0 (0)	0 (0)	-	-	-
Immunodeficiency disease (N, %)	4(1.59%)	3 (0.70%)	0.516	0.473	2.294(0.509,10.335)
Malnourished (N, %)	1(0.40%)	1(0.23%)	< 0.001	1.000	1.708(0.106,27.427)
Abnormal renal function (N, %)	2(0.80%)	7(1.64%)	0.330	0.565	0.483(0.100,2.344)
Anaemia (N, %)	5(1.99%)	6(1.40%)	0.075	0.785	1.430(0.432,4.733)
**Other underlying diseases (N, %)**	**24(9.56%)**	**22(5.14%)**	**4.898**	**0.027**^**a**^	**1.951(1.070,3.558)**
Cerebral complications					
**Hydrocephalus (N, %)**	**35(13.94%)**	**20(4.67%)**	**18.269**	** < 0.001**^**a**^	**3.306(1.863,5.866)**
**Ependymitis (N, %)**	**7(2.79%)**	**2(0.47%)**	**4.866**	**0.027**^**a**^	**6.111(1.259,29.647)**
**Cerebrovascular complications (N, %)**	**80(31.87%)**	**51(11.92%)**	**40.468**	** < 0.001**^**a**^	**3.458(2.330,5.133)**
Cerebral abscess (N, %)	2(0.80%)	2(0.47%)	< 0.001	0.982	1.711(0.239,12.221)
**Encephalomalacia (N, %)**	**25(9.96%)**	**6(1.40%)**	**26.597**	** < 0.001**^**a**^	**7.780(3.146,19.243)**
**Subdural effusion (N, %)**	**36(14.34%)**	**19(4.44%)**	**20.845**	** < 0.001**^**a**^	**3.604(2.019,6.436)**
**Cerebral edema (N, %)**	**12(4.78%)**	**7(1.64%)**	**5.755**	**0.016**^**a**^	**3.020(1.173,7.774)**
**Other cerebral complications (N, %)**	**9(3.59%)**	**4(0.93%)**	**4.594**	**0.032**^**a**^	**3.942(1.201,12.937)**
Bacterial cultures					
Escherichia coli (N, %)	27(10.76%)	29(6.78%)	3.314	0.069	1.658(0.958,2.872)
**Streptococcus agalactiae (N, %)**	**31(12.35%)**	**23(5.37%)**	**10.520**	**0.001**^**a**^	**2.481(1.412,4.360)**
Coagulase-negative Staphylococci (N, %)	13(5.18%)	12(2.80%)	2.518	0.113	1.894(0.850,4.217)
Streptococcus pneumoniae (N, %)	2(0.80%)	3(0.70%)	< 0.001	0.888	1.138(0.189,6.856)
Staphylococcus aureus (N, %)	6(2.39%)	3(0.70%)	2.282	0.131	3.469(0.860,13.996)
Other Gram-positive bacteria (N, %)	2(0.80%)	5(1.17%)	0.005	0.945	0.680(0.131,3.529)
**Other Gram-negative bacteria (N, %)**	**11(4.38%)**	**8(1.87%)**	**3.674**	**0.055**^**a**^	**2.406(0.955,6.065)**
Bacterial species ≥ 2 (N, %)	8(3.19%)	9(2.10%)	0.762	0.383	1.533(0.584,4.025)
Time to start antibiotic therapy (day)	1.0 (0.6,1.4)	0.5 (0.3,0.6)	-1.657	0.098	**-**
**Antibiotic regimens complied with guideline (N, %)**	**172(68.53%)**	**249(58.18%)**	**7.192**	**0.007**^**a**^	**1.565(1.127,2.174)**
Adequate antibiotic treatment course (N, %)	150(72.82%)	184(71.32%)	0.127	0.721	1.077(0.716,1.621)
Adjuvant steroid regimens complied with guideline (N, %)	15(5.98%)	26(6.07%)	< 0.001	0.958	0.983(0.510,1.893)

OR (95%CI): odds ratio and 95% confidence interval; ^**a**^* P* < 0.05

**Table 4:** Factors independently associated with delayed defervescence in BM patients under 3 years of age

	B	S.E	Wald	*P*	Exp (B)	95%Exp (B)	
						Lower limit	Upper limit
**Gastrointestinal infections**	**0.244**	**0.101**	**5.840**	**0.016**^**a**^	**1.276**	**1.047**	**1.556**
Chronic pulmonary diseases	0.264	0.163	2.623	0.105	1.302	0.946	1.791
Congenital heart diseases	0.064	0.041	2.379	0.123	1.066	0.983	1.155
Other underlying diseases	0.031	0.044	0.511	0.475	1.032	0.947	1.124
Hydrocephalus	0.079	0.044	3.229	0.072	1.083	0.993	1.181
Ependymitis	0.170	0.122	1.952	0.162	1.185	0.934	1.504
**Cerebrovascular complications**	**0.159**	**0.037**	**18.800**	** < 0.001**^**a**^	**1.172**	**1.091**	**1.260**
**Encephalomalacia**	**0.292**	**0.131**	**5.005**	**0.025**^**a**^	**1.339**	**1.037**	**1.730**
**Subdural effusion**	**0.374**	**0.106**	**12.469**	** < 0.001**^**a**^	**1.454**	**1.181**	**1.789**
Cerebral edema	-0.229	0.303	0.572	0.450	0.795	0.439	1.441
Other cerebral complications	0.119	0.084	2.007	0.157	1.126	0.956	1.327
**Streptococcus agalactiae**	**0.117**	**0.044**	**7.030**	**0.008**^**a**^	**1.124**	**1.031**	**1.226**
Other Gram-negative bacteria	0.056	0.065	0.741	0.389	1.058	0.931	1.202
Antibiotic regimens complied with guideline	0.178	0.184	0.940	0.332	1.195	0.833	1.714

*BM* bacterial meningitis; ^**a**^*P* < 0.05

## Discussion

In this study, 863 of 900 BM patients diagnosed at the 49 hospitals in Zhejiang province, China, were enrolled, providing reliable and representative data from the region. Most enrolled patients were males (64.7% vs 35.3%). Moreover, most cases had underlying comorbidities, especially congenital heart disease. About 86.6% of the patients had other foci of infection, especially septicaemia. Similar characteristics have been reported in other districts in China and other developing countries [1, 17]. Furthermore, the rate of CSF culture positivity was low (26.2%), similar to other studies (6.7%-24.3%) [18, 19], possibly because antibiotic therapy was given before collection of CSF specimens [5]. *Escherichia coli* was the most common identified pathogen (7.9%), followed by *Streptococcus agalactiae* (7.4%) and coagulase-negative Staphylococci (4.9%). Previous studies showed that these pathogens are the most common organisms causing BM in younger children [20, 21]. Notably, besides positive culture results, clinical manifestations and other tests are needed to indicate the presence of specific pathogens infection. Particularly, some organisms can cause contamination of clinical specimens, such as coagulase-negative staphylococci, and thus the isolates were comprehensively assessed to confirm the relevant source of disease, thus ensuring the accuracy of the results. In this study, about 31.9% of the BM patients developed cerebral complications, especially haemorrhage, and infarction, which are induced by vascular inflammation caused by bacterial virulence factors [22, 23].

Herein, the treatment regimens were reviewed and compared with the comprehensive and authoritative guideline ESCMID [5]. The mean time from admission to antibiotic therapy was 1.0 days, indicating timely therapy administration on the day of admission. Furthermore, choice of antibiotic was acceptable in 61.0% of cases, and duration of administration was acceptable in 48.1%. However, compliance to guidelines for steroid administration was low (5.1%). The non-adherence to guideline management may be due to over-reliance on symptoms, such as fever (present in 79.5% of patients), or delayed treatment in some patients without laboratory test results [24]. Although dexamethasone should be administered before the first dose of antibiotic therapy [5], it is often not feasible in settings where the first antibiotic dose may have been administered before referral to hospital according to Integrated Management of Childhood Illness (IMCI) guidance. In addition, the relevant expert consensus in China currently only includes steroid hormones as part of symptomatic supportive treatment and not used as part of standardized treatment in clinical practice. Steroids are often given as symptomatic treatment according to changes in the child's condition [25]. Notably, the variables of time to initial treatment and the normative application of steroids were included because they positively impact the condition as shown in previous studies. Besides, these high rates of inconsistency with guidelines are not the result of the lack of standardised diagnostic treatment across the multiple centres in this study, but rather the result of benchmarking our results against the latest international guidelines. The results of our analysis challenge clinicians in the region to familiarise themselves with international guidelines, to identify younger pediatric BM patient as early as possible, and to standardize the use of steroids to further improve the treatment of children with BM.

Herein, multivariate analysis suggested that coagulase-negative Staphylococci infection is an independent risk factor for BM patients under 3 years of age with no fever. Besides, previous studies showed that some BM patients with coagulase-negative Staphylococci do not have fever. Nonetheless, no study has compared the fever rates between patients with coagulase-negative Staphylococci infection and patients with infections caused by other pathogens to identify pathogens associated with BM without fever [21]. In this study, a relatively large number of BM patients under 3 years of age with coagulase-negative staphylococci were enrolled, thus providing relatively robust evidence of an association between younger children with BM and this pathogen. Additionally, most BM patients under 3 years of age with septicemia or ENT infection had fever. Although only a few studies have investigated the specific impact of comorbid foci of infection on the BM disease, studies have shown that patients with sepsis alone or bacterial ENT infection experience fever, consistent with this study [26, 27]. These findings show that BM patients under 3 years with fever and afebrile may have concurrent septicaemia or ENT infection.

In this study, 37.0% (251/679) of BM children with fever had delayed defervescence following antibiotic administration. Furthermore, factors that were independently associated with a prolonged course of fever (> = 7 days) were associated with BM in patients under 3 years in concomitant gastrointestinal infection group. In addition, intracranial complications of BM, such as the development of subdural effusions, were associated with delayed defervescence, similar to previous studies [13]. Other intracranial complications, such as encephalomalacia and cerebrovascular complications, were also independently associated with delayed defervescence. Several studies have revealed that brain injury caused by cerebral complications may lead to thermoregulatory dysfunction due to pathological changes in cerebral blood flow, metabolic disturbances, and neurogenic inflammatory responses, leading to the development of temperature instability [28]. In this study, S. *agalactiae* infection was independently associated with prolonged fever. Previous studies also suggested that invasive disease caused by S. *agalactiae* is associated with fever and temperature instability [29, 30]. Therefore, children with BM may have intracranial complications, including subdural effusions, if there is a prolonged course of fever after the administration of appropriate antibiotic therapy.

However, this study has some limitations. First, this is a retrospective study, and thus limits search for factors associated with the main outcomes of interest. Several unknown confounding factors affect observational studies, such as the precision of the collection time in this study regarding the time of start antibiotic therapy only to half a day, when such a time variable would be better precise to the hour. Second, some BM cases were excluded due to incomplete data. Therefore, larger and prospective studies of childhood BM are needed to validate these findings. Third, there were potential quality inconsistencies in the antibiotics used in children. For example, the differences in production batches may impact the study outcome of time to fever resolution. Nonetheless, this is the first and largest study in China to reveal factors independently associated with fever and resolution of fever in BM patients under 3 years of age.

## Conclusions

In conclusion, lack of fever on clinical presentation should not be considered as evidence against BM in patients under 3 years of age, which may also be associated with coagulase-negative staphylococcal infection. Furthermore, BM patients under 3 years of age may have gastrointestinal infection, infection of *Streptococcus agalactiae,* or intracranial complications if there is a prolonged fever after appropriate antibiotic therapy.

### Supplementary Information

**Additional file 1.**

## Data Availability

The data supporting these findings are available from the corresponding author upon reasonable request.

## Abbreviations

BM: Bacterial meningitis
OR: Odds ratios
CSF: Cerebrospinal fluid
ESCMID: European Society for Clinical Microbiology and Infectious Disease
95% CI: 95% Confidence interval
ENT: Ear-nose-throat
