# Supplementary material for: Factors associated with afebrile presentation and delayed defervescence of bacterial meningitis in children under 3 years of age: a multi-centre retrospective analysis
Source: BMC Pediatr. 2023 Sep 18;23:470. doi: 10.1186/s12887-023-04179-8 (PMC10507889; doi:10.1186/s12887-023-04179-8)
Supplement: Supplementary file 1 — Additional file 1. [file 12887_2023_4179_MOESM1_ESM.docx]

There was nine patients with positive bacterial cultures for CoNS were recognized

as “potential contaminant in CSF”. They were only cultured positive in the cerebrospinal fluid and this bacterium was not cultured in the blood, while the clinical features and other laboratory indicators of the child did not meet the criteria for bacterial meningitis. Details of these nine children are as follow: there were 5 males(55.55%), and only 1 case had respiratory infection, 1 case had gastrointestinal infection. A total of 2 cases (22.22%) had underlying diseases, of which 1 cases (11.11%) had congenital heart diseases, 1 cases (1.11%) had anemia. Imaging results and cranial ultrasound showed that no case of nine had cerebral complications.
